# An E2F1/MiR-17-92 Negative Feedback Loop mediates proliferation of Mouse Palatal Mesenchymal Cells

**DOI:** 10.1038/s41598-017-05479-7

**Published:** 2017-07-11

**Authors:** Ling Li, Bing Shi, Jin Chen, Chunhua Li, Shaoxin Wang, Zhaohui Wang, Guiquan Zhu

**Affiliations:** 1 Department of stomatology, Sichuan Cancer Hospital, Sichuan Cancer Center, School of Medicine, University of Electronic Science and Technology of China, Sichuan, China; 2 Department of Head and Neck Surgery, Sichuan Cancer Hospital, Sichuan Cancer Center, School of Medicine, University of Electronic Science and Technology of China, Sichuan, China; 3 State Key Laboratory of Oral Diseases, Department of Cleft lip and Palate Surgery, West China Hospital of Stomatology, Sichuan University, Sichuan, China

## Abstract

Normal cell cycle progression and proliferation of palatal mesenchymal cells are important for palatal development. As targets of miR-17-92, E2F transcription factors family has been suggested to induce the transcription of miR-17-92 in several cell types. In the present study, we sought to investigate whether this negative feedback loop exists in mouse PMCs and what the function of this negative feedback loop would be in palatal mesenchymal cells. Using GeneMANIA, we revealed that the most important function of experimentally verified targets of miR-17-92 is cell cycle regulation. E2F1 and E2F3, but not E2F2, were extensively expressed in mouse palate. Over-expression of E2F1 significantly increased the expression of all the members of miR-17-92. After increased by E2F1, miR-17 and miR-20a may negatively target E2F1, and thereby prevent the cells from excessive proliferation. We suggest that the negative feedback loop between E2F1 and miR-17-92 may contribute to palatal development by regulating the proliferation and cell cycle of palatal mesenchymal cells.

## Introduction

Cleft palate is a common congenital deformity, caused by mal-development of secondary palate. The secondary palate primordium extends from the oral surface of the maxillary processes to form palatal shelves. Before palatal fusion, palatal development includes palatal shelf elongation and elevation. The normal processing of these two steps demands normal proliferation and cell cycle progression of palatal mesenchymal cells (PMCs)^1^. Disturbance of either of the two steps can cause cleft palate.

Normal cell cycle progression and proliferation of PMCs are important for palatal development^2, 3^. In general, cell cycle consists of four distinct phases: G1 phase, S phase, G2 phase, M phase. The transition from G1 to S phase is one of the pivotal processes caused by dynamic and complex interactions of proteins, in which cells become committed to DNA replication. In the G1/S transition, E2F-mediated transcription leads to an accumulation and activation of Cyclin E, Cyclin A, and cyclin-dependent kinase 2 (CDK2), serving as the trigger for S-phase entry^4^.

The family of the E2F transcription factors plays critical role in cell proliferation by controlling the transcription of many of the key components of the cell cycle. This family can be divided into strong transcriptional activators (E2F1/2/3) and repressors (E2F4/5/6/7). The E2F family sets up a network that not only controls cell proliferation, but also participates in checkpoint control, differentiation, and apoptosis. E2F1, a key member of E2F family proteins, acts as a direct executor of G1/S transition^5^ via inducing various genes required for G1/S transition^6^. However, the function of E2F family in palate development has not been well elucidated yet. It has been reported that E2F1 and E2F3 were expressed in palatal tissue of embryonic day (E) 12,13,14 embryos and that E2F4 and E2F5 were highly expressed in palatal tissue on E12 and E13 while decreasing on E14.

MiRNAs are ~22 nucleotide RNAs that regulate post-transcriptional eukaryotic gene expression during embryonic development. MiRNAs can bind to the 3′-UTR of the target mRNAs and thus inhibit their protein translation. In the past decades, the specific biological functions of miRNAs in specific cells or tissues have gained much attention. One of the best characterized polycistronic miRNAs is mir-17-92^7^. The miR-17-92 cluster is conserved among vertebrates, comprising six miRNAs: miR-17, miR-18a, miR-19a, miR-20a, miR-19b-1 and miR-92a-1^8^. Originally found to be an onco-miRNA overexpressing in a variety of malignancies, miR-17-92 cluster has been demonstrated to function in a wide variety of settings, including normal development^8^. MiR-17~92−/− mice exhibit smaller size and die at birth, due to severe lung hypoplasia and cardiac defects^9^. In our previous study, miR-17-92 cluster has been found to continuously express in PMCs and palatal shelves in mouse embryo during E12–14, which is the critical period for palatal shelf elongation and elevation^10^. However, the mechanism by which miR-17-92 modulates palate development has been poorly understood.

An negative regulatory feedback loop between miR-17-92 cluster and E2F family has been described in Hela cells and neural stem cells^11, 12^. This negative feedback loop between miR-17-92 cluster and E2F family is important for preventing an abnormal accumulation of E2F1–3 and may play a role in the regulation of cellular proliferation and apoptosis^11, 12^. Because E2F family plays critical roles in cell cycle regulation, we hypothesized that there may be a negative feedback loop between miR-17-92 cluster and E2F family in PMCs, which may regulate the cell cycle of PMCs and palate development. In this paper, we show that E2F1 was increasingly expressed from E12 to E14 in PMCs. E2F1 induced miR-17-92 expression and proliferation of PMCs. In addition, miR-17 and miR-20a could directly target the 3′UTR of E2F1 in PMCs and imped G1/S transition of PMCs. We thus suggest that the negative feedback loop between miR-17-92 and E2F1 in PMCs plays important roles in regulating the cell cycle transition and proliferation of PMCs during the normal development of palate.

## Results

### Functional analysis of validated miR-17-92 target genes

**Figure 1:**
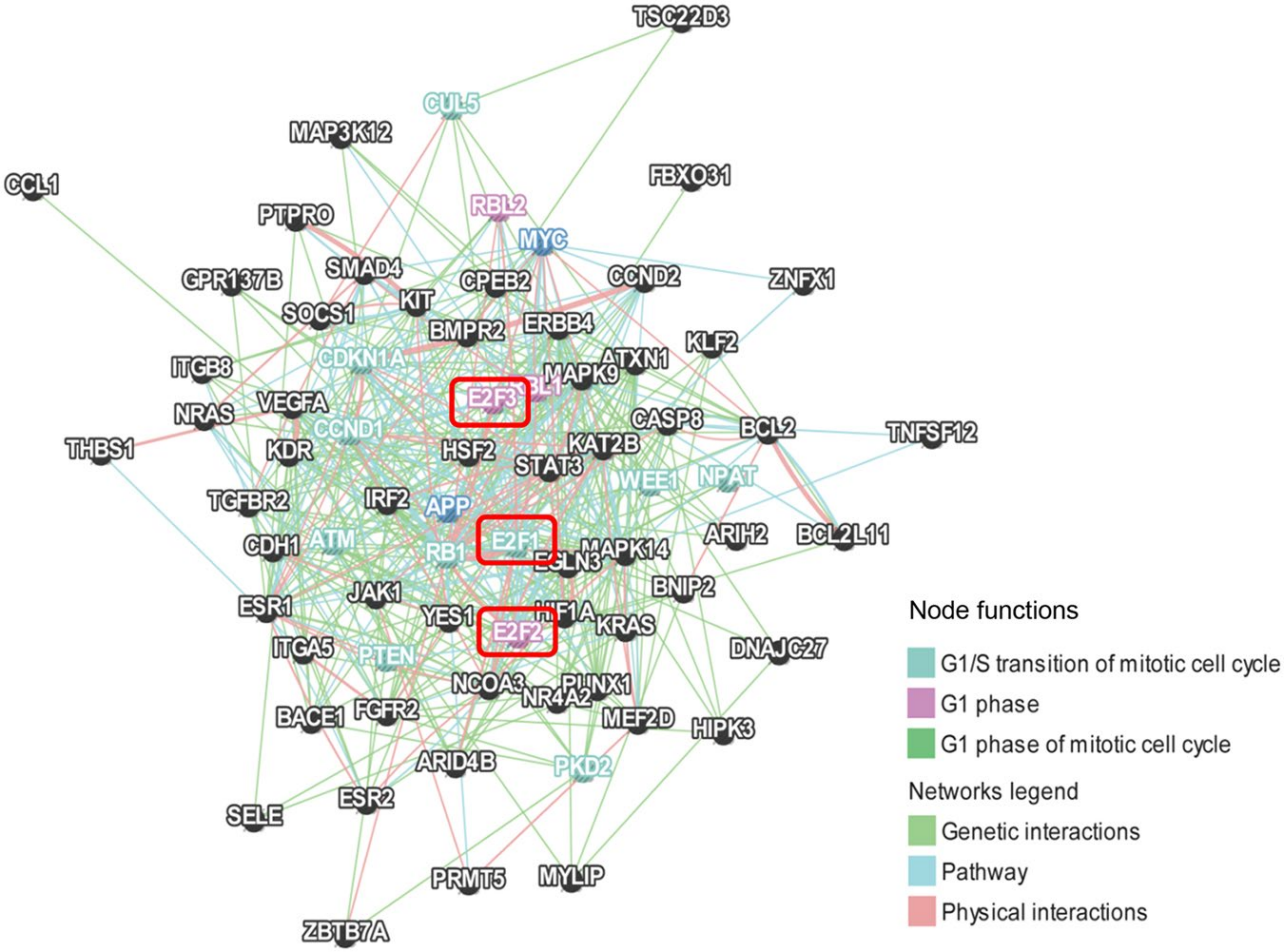
The functions of experimentally verified target genes of miR-17-92 were analyzed by GeneMANIA using genetic interactions, pathway, and physical interaction parameters. The network shows the most outstanding function of these target genes is cell cycle regulation.

To better understand the function of miR-17-92 cluster during palatal development, we identified the experimentally verified target genes of miR-17-92 using the miRTarBase^13^. A total of 91 genes were found to be miR-17-92 targets, among which, 41 of them are targeted by more than one components of miR-17-92. We further analyzed the function of these 91 genes and the functional association between them using GeneMANIA^14^. The most relevant function of these queried genes is the regulation of mitotic cell cycle. The functional associations between miR-17-92 target genes were shown in Fig. 1. As verified targets of miR-17-92, the E2F family, including E2F1, E2F2, and E2F3, plays central role in the functional network.

The sophisticated regulation of cell cycle is important during the embryonic development. However, how developmental cues controlling the cell cycle in different developmental situations remains to be addressed^15^. Because miR-17-92 and E2F family play pivotal roles in the cell cycle control, we hypothesized that the auto-regulatory feedback loop between mir-17-92 and E2F family may regulate palate development via cell cycle modulation.

### E2F1 regulated proliferation of PMCs

**Figure 2:**
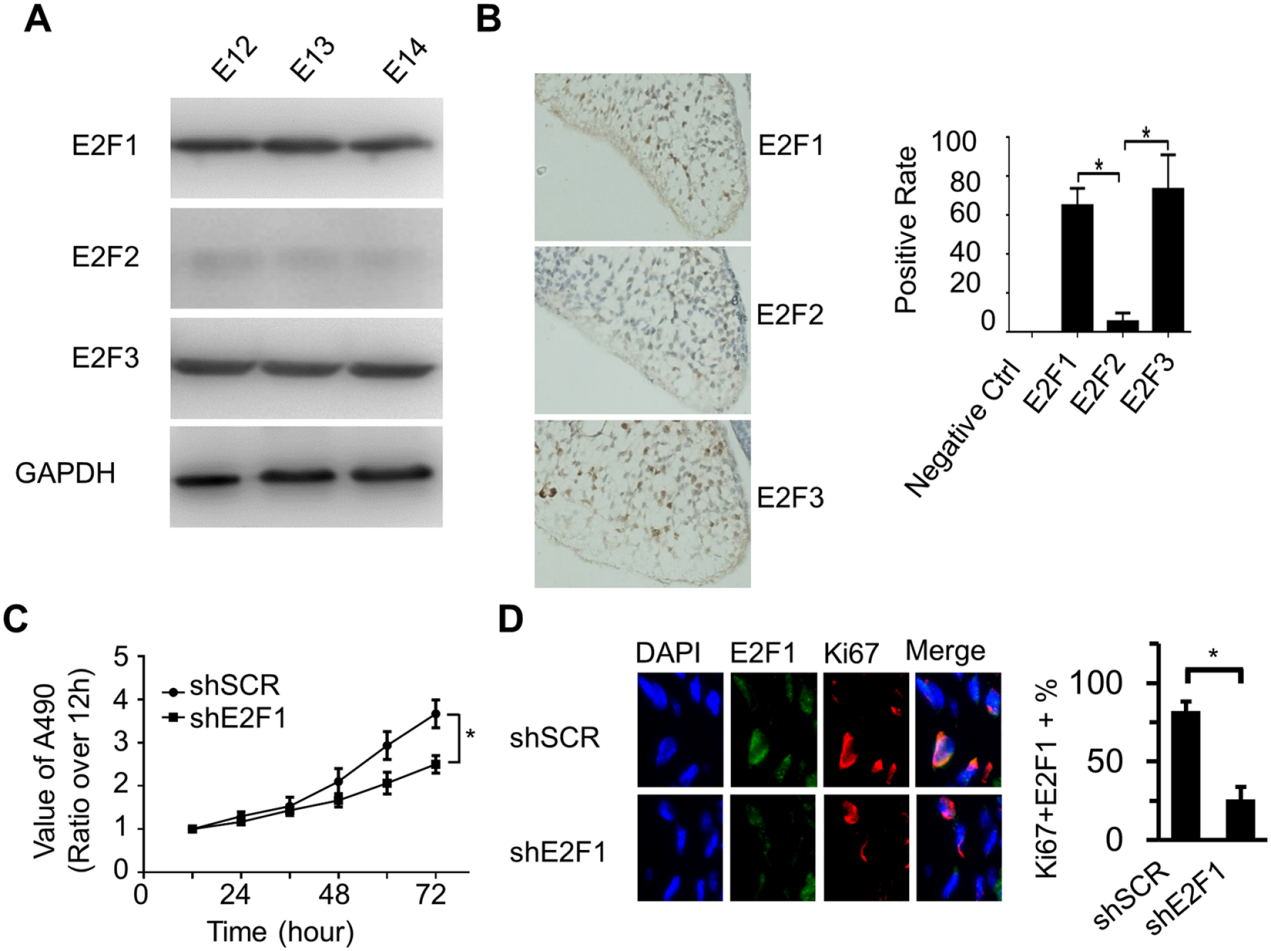
Role of E2F1 in regulating the proliferation of PMCs. (**A**) The expression of E2F1-3 in palatal shelves on E12, E13 and E14 determined by Westernblot; Blots were cropped for figure construction. (**B**) Expression of E2F1-3 on E13.5 was measured by immunohistochemical staining (left panel, magnification ×100). The positive rate of E2F1 and E2F3 in palatal shelves was significantly higher than E2F2 (right panel, n = 6, *P < 0.01). (**C**) MTT assay showing the growth curve of PMCs with or without E2F1 knockdown. Values are mean ± SD of triplicate experiments in each group. *P < 0.01. (**D**) Left panel: immunofluorescence staining of Ki-67 and E2F1 in PMCs. Right panel: the quantification of Ki67 and E2F1 double positive cells. *P < 0.01.

E2F1/2/3 expression during mammal palatal development has not been described in detail. We sought to examine E2F1/2/3 expression from E12 to E14 in palatal shelves of the C57BL/6J mouse. Westernblot showed that E2F1 and E2F3 were strongly expressed in palatal shelves from E12 to E14, while E2F2 having a relatively low levels (Fig. 2A). The expression pattern of E2F1-3 in palate at E13.5 was further evaluated by immunohistochemistry. Approximate 65% of cells had nuclear expression of E2F1 at E13.5 and 75% of cells had nuclear expression of E2F3. E2F2 was expressed in approximate 5% of PMCs (Fig. 2B).

To test whether E2F1 could regulate proliferation of PMCs, shRNA targeting E2F1 (shE2F1) was used to knock down E2F1. MTT assay and immunofluorescence staining of Ki-67 were applied to detect the cell proliferation. The knockdown of E2F1 significantly inhibited the proliferation of PMCs determined by MTT assay. (Figure 2C) Moreover, the percentage of GFP/Ki67 double positive PMCs was significantly decreased by shE2F1 knockdown (Fig. 2D). These results suggest that E2F1 may regulate the proliferation of PMCs during the development of palate.

### Positive regulation of miR-17-92 by E2F1

**Figure 3:**
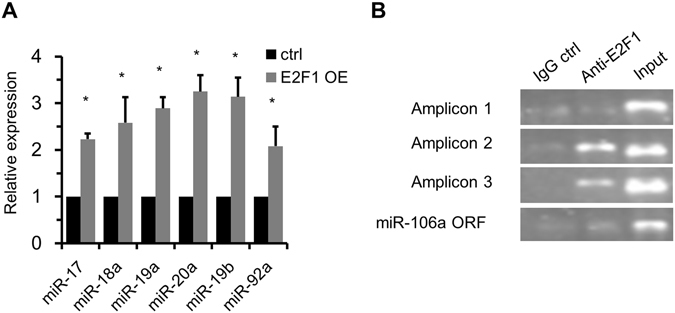
Positive regulation of miR-17-92 by E2F1. (**A**) The expression of miR-17-92 members in control and E2F1 overexpressing cells was analyzed by RT-qPCR. Values represent means ± SD. Error bars, SD. *P < 0.05. (**B**) To detect the direct regulation of E2F1 on miR-17-92, a ChIP assay was performed with 3 putative binding sites of E2F1 on the miR-17-92 promoter. Input DNA that was not enriched by immunoprecipitation was amplified as a positive control. The miR-106a ORF region was used as negative control. The gels were cropped for figure construction.

E2F1 regulates target gene expression usually via binding to the regulatory elements^11, 12^. We sought to investigate whether E2F1 could regulate the expression of miR-17-92 in PMCs. PMCs were transfected with plasmids expressing E2F1 or GFP (control) respectively. Twenty-four hours after the transfection, RT-qPCR was used to measure the expression of miR-17-92 members. We found that over-expression of E2F1 significantly upregulated the expression of all the members of miR-17-92 (Fig. 3A).

Previous studies have shown that miR-17-92 had an E2F1consensus binding sequence on its regulatory elements^11, 12^. Putative E2F1 consensus binding site were identified using “Sitescan” and “TFSEARCH”. We used TTTSSCGC as an E2F1 binding sequence (where S = C or G)^16^, and looked for putative E2F1-binding sites by spanning −3 kb of genomic miR-17-92 cluster. Three putative binding sites were identified to match this canonical sequence. To confirm the direct regulation of E2F1 on miR-17-92, a ChIP assay was performed using PMCs. The miR-106a ORF region was used as negative control^11^. As shown in Fig. 3B, the PCR products of the second and third binding sites were detected when E2F1 was immunoprecipitated. These results validate that E2F1 could direct bind to the promoter of miR-17-92 and promote the transcription of miR-17-92in PMCs.

### Negative regulation of E2F1 by miR-17-92

**Figure 4:**
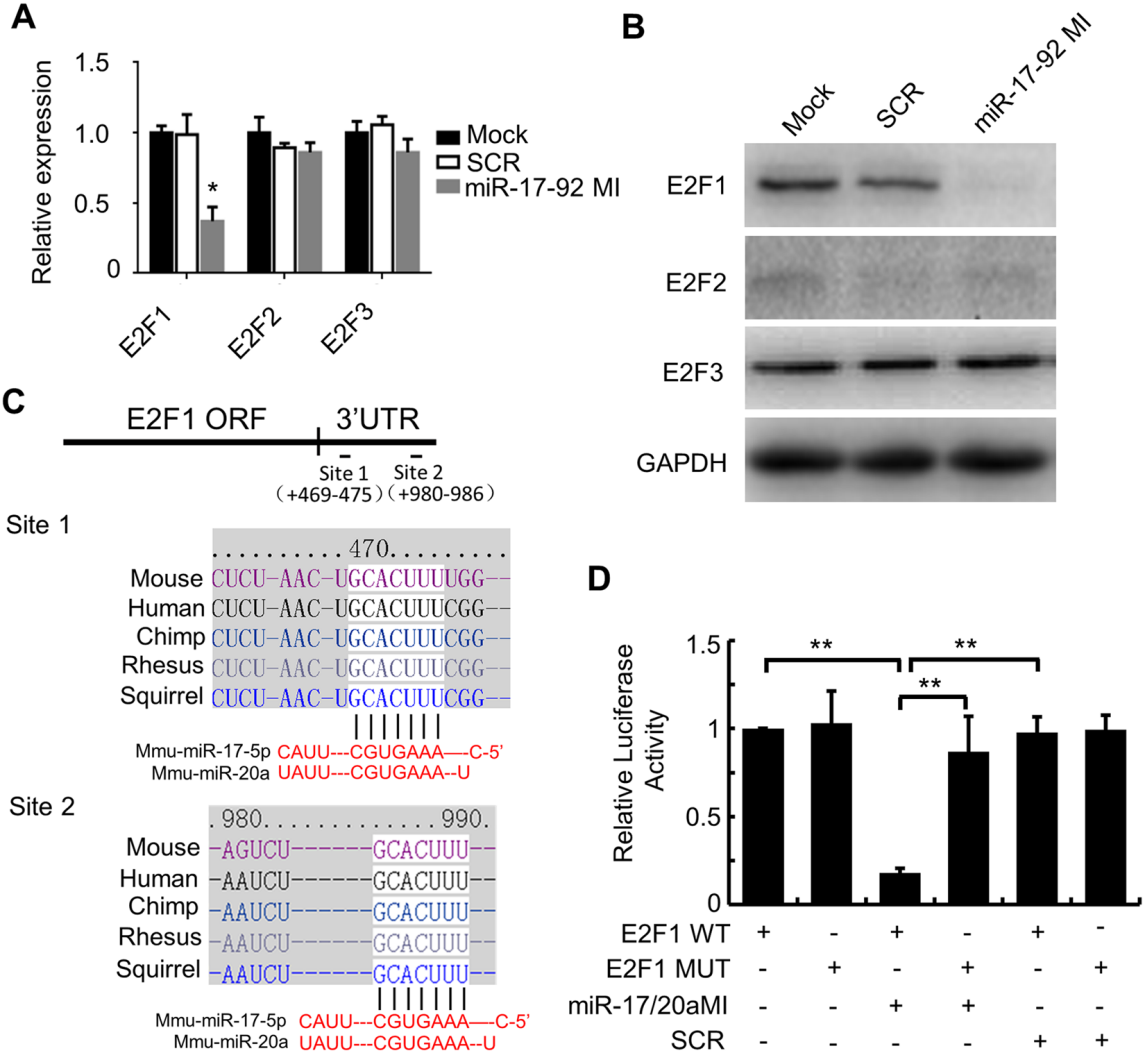
Negative regulation of E2F1 by miR-17-92. (**A**) The relative expression of E2F1-3 mRNA was evaluated by qPCR. Values represent means ± SD. Error bars, SD. *P < 0.05. (**B**) Protein levels of E2F1-3 were measured by Western blot analysis. GAPDH was used as the internal control. Blots were cropped for figure construction. (**C**) The location and sequences of predicted target sites in the 3′UTR of mouse E2F1 for miR-17/20a. The seed sequences of miR-17/20a. The sequences of predicted target sites are conserved across species including human and mouse. (**D**) Luciferase assay was used to validate the predicted target sites of miR-17/20a on 3′UTR of E2F1. Data were presented as relative luciferase activities compared to normoxia after normalizing to Renilla luciferase activities. Experiments were performed in triplicate (*P < 0.05).

To investigate whether E2F1-3 in mouse PMCs could be targeted by miR-17-92, primary cultured PMCs were transfected with miR-17-92 mimics. The expressions of E2F1-3 in mRNA and protein levels were evaluated by qPCR and Westernblot respectively. Both mRNA (Fig. 4A) and protein (Fig. 4B) levels of E2F1 were significantly decreased by miR-17-92 mimics. Either E2F2 or E2F3 was influenced by miR-17-92 mimics. Our results suggest that, in PMCs, only E2F1 is targeted by miR-17-92.

We next investigated whether mir-17-92 could directly down-regulate E2F1 in PMCs. Using the software ‘TARGETSCAN’, “PICTAR” and ‘MIRANDA’, we found that miR-17 and miR-20a have the same seed sequences and two predicted target sites in the same region of the 3′UTR of mouse E2F1 (Fig. 4C). However, we didn’t find predicted target site of miR-18a/19a/19b/92a on E2F1. Thus we generated pMIR-Report luciferase vectors of E2F1 for miR-17/20a. The coding sequence of the firefly luciferase is followed by ~100 nucleotide synthetic DNA fragments encompassing the predicted miRNA binding site from predicted target gene 3′-UTR in either wild-type (WT) or seed mutant constructs. The 3′UTR recombinant construct of E2F1 was transfected into PMCs along with miR-17/20a mimics or scrambled miRNAs. The luciferase activity in PMCs transfected with E2F1 WT constructs plus miR-17/20a mimics was significantly lower than that in PMCs transfected with either E2F1 WT or mutant constructs alone, and the scrambled miRNA did not affect the luciferase activity in either WT or mutant constructs transfected PMCs (Fig. 4D). This result indicates that E2F1 is a direct target of miR-17 and miR-20a in PMCs.

### MiR-17-92 negatively regulates E2F1-induced cell cycle transition of PMCs

**Figure 5:**
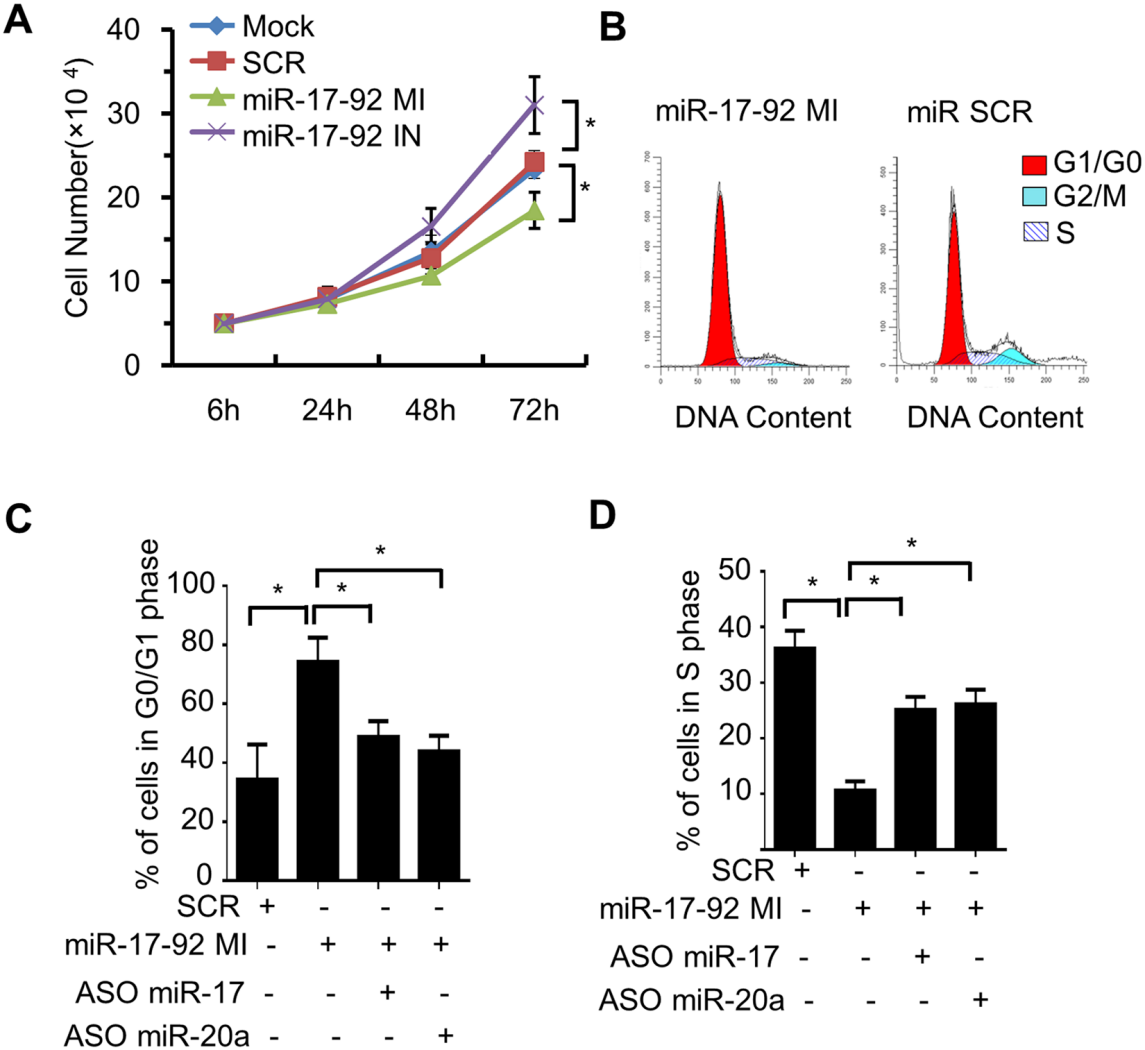
MiR-17-92 negatively regulates E2F1-induced cell cycle transition of PMCs. (**A**) MTT assay showing the role of miR-17-92 on the growth of PMCs. Values are mean ± SD of triplicate experiments in each group. *P < 0.01. (**B**) Cell cycle of PMCs transfected with miR-17-92 MI or miR SCR was analysed by flow cytometry. (**C**) Quantification analysis of cells in G0/G1 phase. Values are mean ± SD of triplicate experiments in each group. *P < 0.01. (**D**) Quantification analysis of cells in S phase. Values are mean ± SD of triplicate experiments in each group. *P < 0.01.

To investigate whether miR-17-92 could regulate cell cycle of PMCs through targeting E2F1, PMCs were transfected with miR-17-92 mimics, miR-17-92 inhibitors, and scrambled miRNAs respectively. The proliferation rate was measured up to 72 h after transfection using MTT assay. We found that PMCs transfected with miR-17-92 mimics had a significantly lower cell proliferation rate than that transfected with scrambled miRNAs. Cells transfected with miR-17-92 inhibitors had a significantly higher proliferation rate compared with those transfected with scrambled miRNAs (Fig. 5A).

Flow cytometry analysis was used to examine the cell cycle of PMC with PI staining (Fig. 5B). PMCs transfected with miR-17-92 mimics had a higher rate of G0/G1 phase (Fig. 5C) while lower rate of S phase compared with those transfected with scramble miRNA (Fig. 5D). To investigate whether this effect was dependent on miR-17 and miR-20a, the same cells were co-transfected with miRNA inhibitors against miR-17 and miR-20a respectively. The inhibition of miR-17 and miR-20a in cells transfected with miR-17-92 mimics decreased the proportion of cells in G0/G1 phase (Fig. 5C) while increasing the percent of cells in S phase (Fig. 5D). These results suggest that the effect of miR-17-92 on cell cycle control is dependent on miR-17 and miR-20a which directly target E2F1 in PMCs.

## Discussion

MiR-17-92 has been found to affect palatal development^17, 18^. In the present study, using GeneMANIA, we revealed that the most important function of experimentally verified targets of miR-17-92 is cell cycle regulation. The functional network provides a framework in which large datasets are analyzed with an unbiased view and their functions are better understood^19^. Also, the network framework is a powerful concept and tool for revealing molecular mechanisms and predictive biomarkers^20^. With the network framework, McGee *et al*. demonstrated a signaling regulatory loop in PIK3CA-mutated breast cancer having a predictive power for the survival of the PIK3CA mutated luminal A patients^20^. In addition, signaling network analysis of ubiquitin-mediated proteins suggests correlations between the 26S proteasome and tumor progression^21^. Our functional network of verified targets of miR-17-92 revealed that E2F family, including E2F1, E2F2, and E2F3, plays a central role in the framework of miR-17-92 target genes.

**Figure 6:**
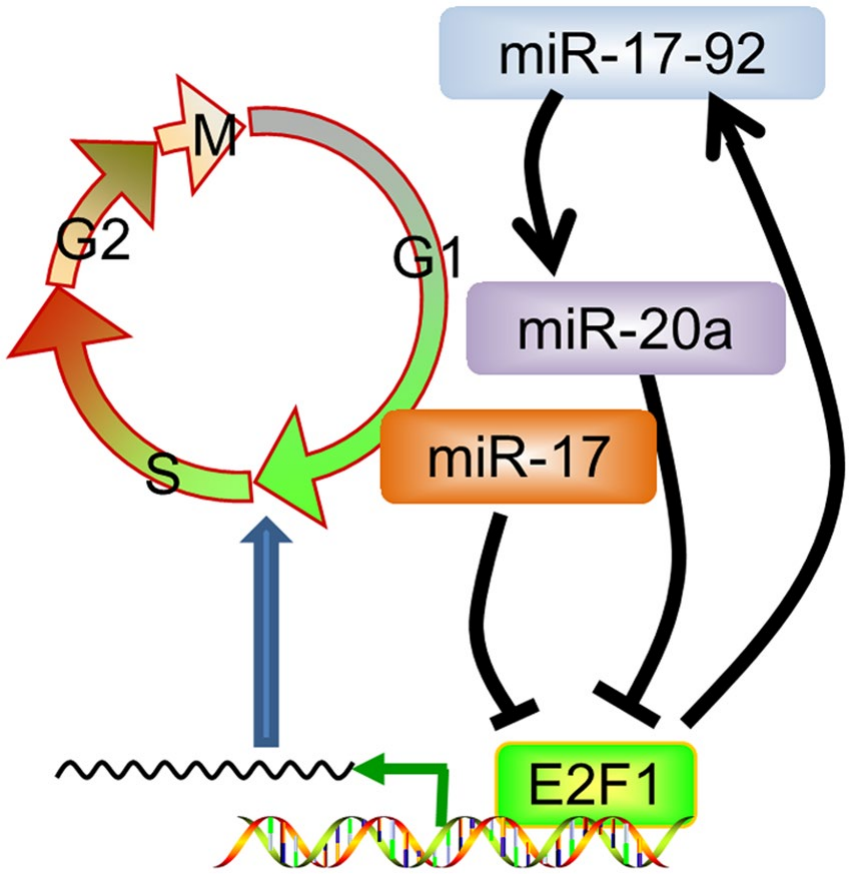
Schematic cartoon illustrating the negative feedback loop between E2F1 and miR-17-92 and its regulation on the cell cycle of PMCs.

As a target of miR-17-92, E2F1 has been suggested to induce the transcription of miR-17-92 in several cell types. We sought to investigate whether this negative feedback loop exists in mouse PMCs and what the function of this negative feedback loop would be in PMCs. Our results showed that E2F1 and E2F3, but not E2F2, were extensively expressed in mouse palate. E2F1 could induce the proliferation of PMCs and the expression of miR-17-92. After increase, miR-17 and miR-20a may negatively target E2F1, and thereby prevent the cells from excessive proliferation. We suggest that the negative feedback loop between E2F1 and miR-17-92 may contribute to palatal development by regulating the proliferation and cell cycle of PMCs (Fig. 6).

In the process of palate development, the secondary palate primordium extends from the oral surface of the maxillary processes to form palatal shelves. Before palatal fusion, palate development includes palatal shelf elongation and elevation. The normal process of these two steps demands normal cell cycle progression of PMCs. Here we found that E2F1 expressed in palate during E12 to E14. Moreover, E2F1 induces proliferation of PMCs *ex-vivo*. Our results suggest that E2F1 in PMCs may contribute to the continuous growth of palate during E12 to E14. E2F2 and E2F3 have been found to express in palatal shelves. In this study, however, the E2F2 expression level was extremely low. The reason might be that E2F1 and E2F2 function at different stage of palate development.

It is well known that E2Fs transcription factors are activated by hypo-phosphorylation and gradual inactivation of Rb by cyclins and CDKs. This process triggers a positive feedback loop. More cyclin/CDK would result in hyper-phosphorylation and complete inactivation of Rb, which activates more E2Fs. This positive feedback mechanism commits a cell to pass the G1/S transition and get into S phase^22^. However, once the palatal shelves are fused, the high proliferation nature of PMCs would be terminated, and the anterior portion undergoes ossification to form the hard palate^23^. Therefore, the activity of E2F1 is needed to be balanced by a negative signal. Mir-17-92, directly induced by E2F1^12, 24^, may act as the negative signal by directly target E2F1. This negative regulation between E2F1 and miR-27-92 may precisely maintain the cell cycle transition and proliferation of PMCs during the development of palate. Abnormal regulation of this negative feedback may result in developmental disorder of palate.

## Materials and Methods

### Cell culture

All the animals involved in our study were approved by the Animal Care and Use Committee of Sichuan Cancer Hospital. All of the experimental procedures followed by Guide for the Care and Use of Laboratory Animals: Eighth Edition (NIH, Bethesda, MD, USA). We dissected palatal shelves from C57BL/6J mouse embryos on E13.5, and separated the mesenchyme from the epithelia. To dissociate individual cells, the palatal mesenchyme was incubated in PBS with 0.25% trypsin and 0.02% EDTA for 15 min. Primary culture of PMCs was initiated by seeding 5 or 2 ml DMEM/F12 into 25 ml flask or flat plate.

### Quantitative real-time RT-PCR

Reverse transcription and qPCR of the components of miR-17-92 cluster was performed using Taqman Small RNA Assay (Applied Biosystems, Foster City, CA, USA) according to the manufacturer’s instructions. Briefly, 10 ng total RNA was reverse transcribed with miRNA specific primers in 15 ml reaction volumes. Reverse transcription reactions were diluted and amplified in triplicates by TaqMan qPCR on a 7300 Real Time PCR System (Applied Biosystems). Quantification was performed using the ΔΔCt method. The RT-PCR fold changes were normalized to snoRNA135 (Applied Biosystems).

For E2F1/2/3, quantitative real-time RT-PCR was carried out as described previously^25^. Briefly, total RNA was isolated with TRIzol reagent (Invitrogen, Carlsbad, CA, USA), and reverse-transcribed using a RevertAid First-Strand cDNA Synthesis Kit (Fermentas). PCR amplification of the cDNA template was done using Thunderbird SYBR qPCR mix (Toyobo, Osaka, Japan) on ABI PRISM 7300 sequence detection system (Applied Biosystems). Reactions were run in triplicate, and results were averaged. Each value was normalized to b-actin. The relative expression level of the genes was calculated using the DDCt method. The sequences of PCR primers were as follows: E2f1: F5′-CTGCAGCAACTGCAGGAGAG-3′; R5′-CTCCGAAAGCAGTTGCAGC-3′; E2F2: F5′-ACGGCGCAACCTACAAAGAG-3′; R5′-GTCTGCGTGTAAAGCGAAGT-3′; E2F3: F5′- GGTCCTGGATCTGAACAAGGC-3′; R5′-CCTTCCAGCACGTTGGTGAT-3′.

### Cell transfection

PMCs were transfected with mixed miRNA mimics of miR-17-92 components, mixed miRNA inhibitors of miR-17-92 components, or scrambled miRNAs (Ribobio, Guangzhou, China) at a concentration of 50nM using Lipofectamine 2000 (Invitrogen, Carlsbad, CA, USA) following the manufacture’s protocol. Total RNA and protein were isolated from the transfected cells, followed by Real-time PCR and Western blot.

Vectors of shE2F1 (Origene TG509487), shSCR (vectors against a scrambled sequence, negative control (Origene TR30013), and E2F1 expression vectors (Addgene plasmid 10736) were transfected into PMCs with TurboFectin 8.0 (Origene, Rockville, MD, USA) following the manufacturer’s protocol. After 6 h of transfection, the medium was replaced by serum-containing medium.

### Western blot

Western blots of E2F1, E2F2 and E2F3 were carried out as described previously^26^. Briefly, total proteins were isolated from the PMCs. Thirty-microgram proteins from each sample were separated on SDS–PAGE and transferred to polyvinylidene difluoride membranes (Millipore, Darmstadt, Germany). Membranes were incubated with anti-E2F1 (Santa Cruz, Dallas, TX, USA), anti-E2F2 (Santa Cruz) and anti-E2F3 (Abcam, Cambridge, MA, USA) antibodies.

### MTT assay for cell proliferation

The cell proliferation was quantified by the colorimetric MTT assay as previous described^27^. In brief, cells were incubated with MTT for 4 h. Then supernatant was removed and DMSO was added. Optical densities at 490 nm were measured using culture.

### Immunohistochemical staining

Sections (4 μm) were deparaffinized in xylene and rehydrated, and endogenous peroxidase was blocked with 3% H_2_O_2_. Antigen retrieval was accomplished by 0.01 mol/L citrate buffer solution (pH 6.0) in a 700W microwave oven for 15 minutes. After incubation with 5% normal goat serum for 20 minutes, the slides were exposed for 1 hour at 37 °C and overnight at 4 °C to the rabbit anti-E2F1(1:200; Santa Cruz), mouse anti-E2F2 (1:200; Santa Cruz), rabbit anti-E2F3(1:200; Abcam). Sections were then incubated with biotinylated goat anti-rabbit IgG/goat anti-mouse IgG (Zhongshan Goldenbridge Biotechnology) for 1 hour, and streptavidin-peroxidase for 30 minutes. The 0.02% diaminobenzidine tetrahydrochloride was used as a chromogen, and the slides were counterstained with hematoxylin.

### Immunofluorescence

PMCs were seeded onto coverslips at a density of 10^4^/mL and cultured in a 6-well culture plate for 24 hours. Cells grown on coverslips were washed in cold PBS and fixed in 2% paraformaldehyde-PBS for 20 minutes, permeabilized in 0.5% Triton X-100 in PBS for 10 minutes at 4 °C, and blocked in 1% bovine serum albumin for 30 minutes at room temperature. Cells were incubated overnight with 1:100 dilution of rabbit anti-E2F1(Santa Cruz), or 1:100 dilution of mouse anti-Ki67 (Santa Cruz), and then incubated with FITC-conjugated goat anti-rabbit IgG (1:500; Zhongshan Goldenbridge Biotechnology) and Alexa Fluor594-conjugated goat anti-mouse IgG (1:200; Zhongshan Goldenbridge Biotechnology) at 37 °C for 1 hour. Cells were counterstained with 4′, 6-diamidino-2-phenylindole (DAPI; 1 μg/μL), and examined using a fluorescence microscope (Olympus BX51). The photographes was analyzed by Image-Pro Plus 4.5 software (Media Cybernetics Inc, Silver Spring, MD) to determine the cell percentage of double-labeled (Ki67+, GFP+) cells/GFP+ cells.

### Luciferase reporter constructs and assays

The two sites of 3′UTR of E2F1 gene were cloned into the pMIR-Report luciferase vector respectively (Ambion). The sense and antisense strands of the oligonucleotides were annealed according to manufacturer’s protocol. The annealed oligonucleotides were digested with HindIII and SpeI and ligated into pMIRReport luciferase vector. A BlpI site was added into each insert to test for positive clones as described^18, 28^. pMIR-Report bgal vector was used as a control for transfection efficiency. PMCs were lysed 24 h after transfection, and reporter activity was measured using Luciferase and b-galactosidase assay kits (Beyotime). Each experiment was repeated at least three times. The sequences of E2F1 3′UTR oligonucleotides were follows: Site 1: E2f1 wide type sense: AATGCACTAGTGGGTGGGCTCTAACTGCACTTTT GCTCAGCAAGCTTAATGC; E2F1 wide type antisense: GCATTAAGCTTGCTGAGCAAAAGTGCAGTTAGAGCCCACCC ACTAGTGCATT; E2F1 mutant sense: AATGCACTAGT GGGTGGGCTCTAACTGGAGTGTT GCTCAGCAAGCTTAATGC; E2F1 mutant antisense: GCATTAAGCTTGCTGAGC AACACTCCAGTTAGAGCCCACCC ACTAGTGCATT. Site 2: E2f1 wide type sense: AATGCACTAGT CCCACCCTCCAGTCTGCACTTTGA GCTCAGCAAGCTTA; ATGC; E2F1 wide type antisense: GCATTAAGCTTGCTGAGC TCAAAGTGCAGACTGGAGGGTGGG ACTAGTGCATT. E2F1 mutant sense: AATGCACTAGT CCCACCCTCCAGTCTGGAGTGTGA GCTCAGCAAGCTTAATGC; E2F1 mutant antisense: GCATTAAGCTTGCTGAGC TCACACTCCAGACTGGAGGGTGGG ACTAGTGCATT.

### Flow cytometry

PMCs transfected with miR-17-92 mimics/inhibitors/scramble were synchronized at the G0/G1 phase of the cell cycle by serum starvation for 24/48 h with 0.5% FBS^29^. Then cells were harvested and stained with propidium iodide (PI) for cell cycle analysis using Click-iT EDU flow cytometry assay kit (Invitrogen, CA, USA) on a Cytomics FC 500 MPL Flow Cytometer (Beckman Coulter, Brea, CA, USA) with RXP software (Beckman Coulter). Data were analyzed using MODFIT LT 4.1 software.

### Chromatin Immunoprecipitation (ChIP) Assay

ChIP assay was conducted with a ChIP assay kit (Upstate Biotechnology, Waltham, MA, USA) according to the manufacturer’s instructions. Briefly, cells were fixed with formaldehyde, lysed, and sonicated to obtain DNA fragments in a size from 200 to 1000 bp. Chromatin was then precipitated with non-specific IgG antibodies (Sigma, St. Louis, MO, USA) or mouse anti-E2F1 (sc-193x, Santa Cruz). DNA was extracted by phenolechloroform and precipitated by ethanol. PCR was performed with primers for three miR-17-92 promoter fragments (Promoter 1F: ACATGGTCCTTCGAGGTGC, Promoter 1R: CCCCACCCCTCGGCCTCG; Promoter 2F: TCACAGCAGTTGGGGAAACA, Promoter 2R: CTCCCCCAATCAGGACCTC; Promoter 3F: GCGGGCCGGGTGGGTCTC, Promoter 3R: GCCAGGACGGCCGCCCCA), and for a fragment containing miR-106a ORF (106a ORF F: CCACAATCAGTTTTGCATGG, 106a ORF R: TTTTGCAGATTTGCAGTTCA) at 55′C for 35 cycles.
